# MiR-205 Mediated Cu-Induced Lipid Accumulation in Yellow Catfish *Pelteobagrus fulvidraco*

**DOI:** 10.3390/ijms19102980

**Published:** 2018-09-29

**Authors:** Heng-Yang Cui, Qi-Liang Chen, Xiao-Ying Tan, Dian-Guang Zhang, Shi-Cheng Ling, Guang-Hui Chen, Zhi Luo

**Affiliations:** 1 Laboratory of Nutrition and Feed Formulation for Aquatic Economic Animals, Fishery College, Huazhong Agricultural University, Wuhan 430070, China; cuihengyang@webmail.hzau.edu.cn (H.-Y.C.); xncql@126.com (Q.-L.C.); txy7933@mail.hzau.edu.cn (X.-Y.T.); ZDG@webmail.hzau.edu.cn (D.-G.Z.); 15907174014@webmail.hzau.edu.cn (S.-C.L.); cgh0026@webmail.hzau.edu.cn (G.-H.C.); 2 Chongqing Key Laboratory of Animal Biology, School of Life Sciences, Chongqing Normal University, Chongqing 401331, China; 3 Collaborative Innovation Center for Efficient and Health Production of Fisheries in Hunan Province, Changde 415000, China

**Keywords:** *Pelteobagrus fulvidraco*, Cu, miR-205, lipid accumulation, lipid metabolism

## Abstract

The present working hypothesis is that the Cu-induced changes in lipid metabolism may be mediated by miRNAs. Here, we describe the miRNA profile of the liver tissues of yellow catfish exposed to waterborne Cu, based on larger-scale sequencing of small RNA libraries. We identified a total of 172 distinct miRNAs. Among these miRNAs, compared to the control, mRNA expression levels of 16 miRNAs (miR-203a, 205, 1788-3p, 375, 31, 196a, 203b-3p, 2187-5p, 196d, 459-3p, 153a and miR-725, and two novel-miRNAs: chr4-1432, chr-7684) were down-regulated, and mRNA levels of miR-212 and chr20-5274 were up-regulated in Cu-exposed group. The functions of their target genes mainly involved ether lipid metabolism, glycerophospholipid metabolism, linoleic acid metabolism and α-linolenic acid metabolism. Cu exposure inhibited the expression of miR-205, whose predicted target genes were enriched in the pathway of lipid metabolism, including *fas*, *lxrα*, *ddit3*, *lamp2*, *casp3a* and *baxa*. These potential target genes were further verified by Dual-luciferase reporter gene assay. Using primary hepatocytes of yellow catfish, Cu incubation down-regulated miR-205 expression, and increased TG contents and FAS activity. LXR antagonist effectively ameliorate the Cu-induced change of TG content and FAS activity. These data suggest that down-regulation of the miRNA-205 may be an important step in Cu-induced changes in lipid metabolism in yellow catfish.

## 1. Introduction

During environmental stress, maintenance of homeostasis requires constant metabolic adjustment, which is achieved partly through the fine-tuning of gene expression. Study showed that microRNAs (miRNAs) responded to environmental stress in fish [1]. miRNAs are highly conserved short non-coding RNAs that repress gene expression by binding to the 3′UTR of target genes [2]. A given miRNA can target a multitude of different mRNAs and a given mRNA might be targeted by many miRNAs, indicating that the relationship between miRNA and their target genes was very complex. At present, in mammals, hundreds of miRNAs have been identified and thousands of targets have been predicted [3,4]. Some studies showed that miRNAs regulate lipid metabolism, including adipocyte differentiation, cholesterol metabolism and high-density lipoprotein (HDL) biogenesis [5,6,7]. In teleosts, miRNAs, along with their associated target genes, are involved in various physiological processes including development, growth and stress response [8,9,10]. Their role in lipid metabolism has also been studied in several fish species, including zebrafish (*Danio rerio*) [11], rainbow trout (*Oncorhynchus mykiss*) [12] and blunt snout bream (*Megalobrama amblycephala*) [13]. However, any further mechanistic relationship between miRNAs and their target genes remains largely unknown.

Cu is a cofactor of a variety of enzymes and plays an important role in fish metabolism; however, it can exert adverse toxicological effects when present in excess [14,15,16,17]. Recently, our studies have pointed out that waterborne Cu exposure could influence lipid metabolism in yellow catfish *Pelteobagrus fulvidraco*, a widely cultured freshwater teleost [18,19,20]. Environmental chemicals such as heavy metals can interfere with the biogenesis and expression of miRNAs, leading to toxicological consequences [21]. miRNA is an important regulator in lipid metabolism. Thus, we hypothesized that certain miRNAs mediated the Cu-induced changes in lipid metabolism in yellow catfish by targeting the genes related to lipid metabolism.

Here, we first used high-throughput sequencing technology to detect and quantify miRNA expression profiles in yellow catfish exposed to waterborne Cu. We found that the expression of miR-205 was markedly down-regulated following Cu exposure. We then used bioinformatics methods to predict target genes of these miRNAs and found that many target genes of miR-205 involved the lipid metabolism. Luciferase reporter gene was used to validate these predicted target genes. We found that miR-205 influenced lipid metabolism by targeting *lxra*, which encodes LXR, a transcription factor that plays an important regulatory role in lipid metabolism. Our findings provide a better understanding for the role of miR-205 in yellow catfish lipid metabolism and may help to identify novel targets for interventions to reduce the occurrence of fatty liver disease in yellow catfish farming.

## 2. Results

### 2.1. Identification of Hepatic miRNAs from Yellow Catfish

**Figure 1:**
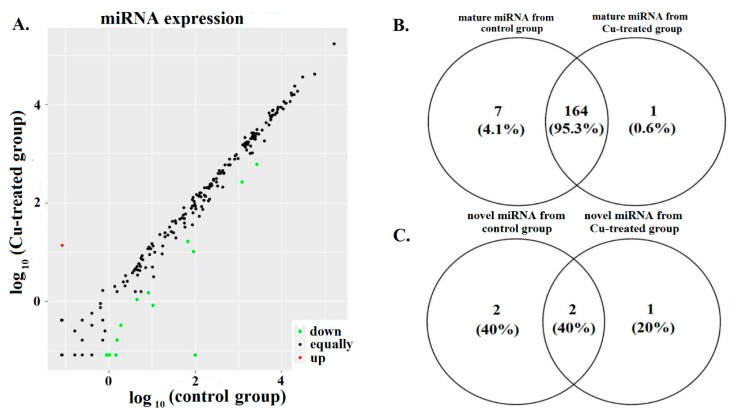
miRNA expression between the control and Cu-exposed group. (**A**) Scatter plot of hepatic miRNA expression levels in control group and Cu-exposed group. Each point represents a miRNA. The X and Y axes showed the normalized expression (log_10_NE) of miRNAs in each liver tissue. (**B**,**C**) Venn diagram compared the expression distribution of mature miRNAs and novel miRNAs between two groups. Numbers represents number and percent of co-expressed or differentially expressed miRNAs.

In this study, length distribution analysis revealed that the most common sequence length in both yellow catfish libraries was 22 nt (Supplementary Figure S1). The 22 nt peak observed in the length distribution in both libraries in this study implies that miRNAs were identified in yellow catfish liver tissue. As shown in Figure 1A and Table S2, there were 172 sequences mapped to the miRNAs and pre-miRNAs of selected species in the miRBase, and these pre-miRs were further mapped to the *Danio rerio* genome. Totally, we identified 171 mature miRNAs in the control and 165 mature miRNAs in the Cu-treated group. Among these mature miRNAs, 164 miRNAs were co-expressed in both the control and Cu-treated group. We also found 4 novel miRNAs (without significant matches against miRBase) in the control and 3 novel miRNAs in the Cu-treated group, which have not been described in previous published miRNA studies. Among these novel miRNAs, 2 novel miRNAs were existent in the control and Cu-treated group (Figure 1B,C). Novel chr20-5274 miRNA was only expressed in the liver of Cu-treated group, and novel chr4-1432 miRNA had high expression in the control and had no expression in Cu-treated group. These results indicated that the spectrum of expressed miRNAs was very different between the control and Cu-exposed group.

**Figure 2:**
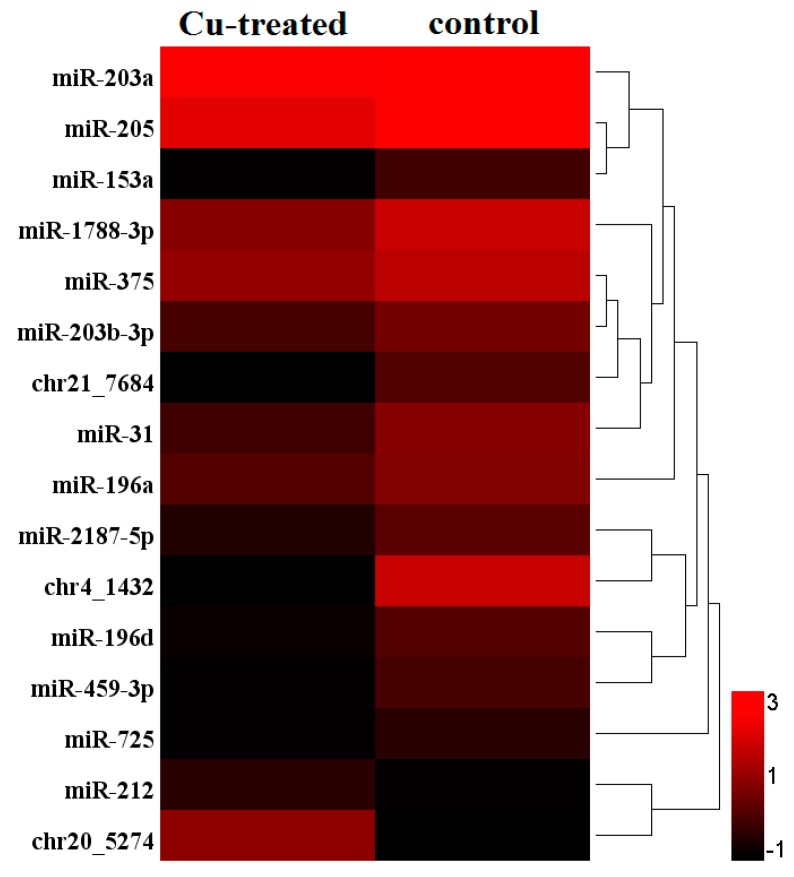
Hierarchical clustering of differentially expressed miRNAs between the two groups. The heat map was drawn with log_10_NE of each miRNA. Color from dark to light indicate low frequency to high frequency miRNA between the control and Cu-exposed group. Color map was used to distinguish the difference of expression.

We further compared the transcriptional level of co-expressed miRNAs between the control and Cu-treated group. In total, 16 miRNAs were differentially expressed between the control and Cu-treated group (Figure 2). Compared to the control, 2 miRNAs (miR-212 and chr20_5274) were up-regulated and 14 miRNAs (miR-203a, miR-205, miR-153a, miR-1788-3p, miR-375, miR-203b-3p, chr21_7684, miR-31, miR-196a, miR-2187-5p, chr4_1432, miR-196d, miR-459-3p and miR725) were down-regulated.

### 2.2. Predicted Targets of Differentially Expressed miRNAs between Two Groups

**Figure 3:**
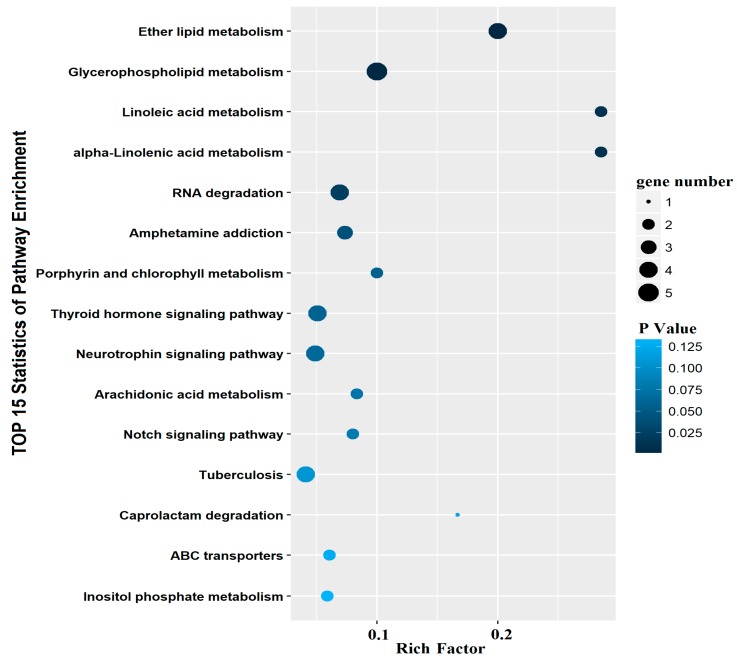
The top 15 KEGG pathway analysis of known miRNAs with differential expression. The horizontal coordinates was the rich factor. The bigger the rich factor the higher the enrichment.

We first used an in silico approach to identify the predicted miRNAs targets by using TargetScan, the database of conserved 3′UTR miRNA targets. The result show that 16 differentially expressed miRNAs had 457 targets, and miR-205 had 42 targets (Table S3). We analyzed the distribution of functional annotation for all targets of differentially expressed miRNAs using GO terms and KEGG. Most of targets were enriched in Biology Process (BP) and the largest number of targets in cell, cell part and binding (Figure S2). Results of KEGG annotation showed that targets of differentially expressed miRNAs were rich in ether lipid metabolism, glycerophospholipid metabolism, linoleic acid metabolism, alpha-linolenic acid metabolism, RNA degradation and amphetamine addiction (Figure 3). Since most of the targets of miR-205 were enriched in the pathway of the lipid metabolism, we speculated that miR-205 mediated Cu-induced changes in the lipid metabolism. Therefore, in the following studies, we place special emphasis on miR-205.

### 2.3. miR-205 Targets fas, lxrα, ddit3, casp3a, baxa and lamp2

**Figure 4:**
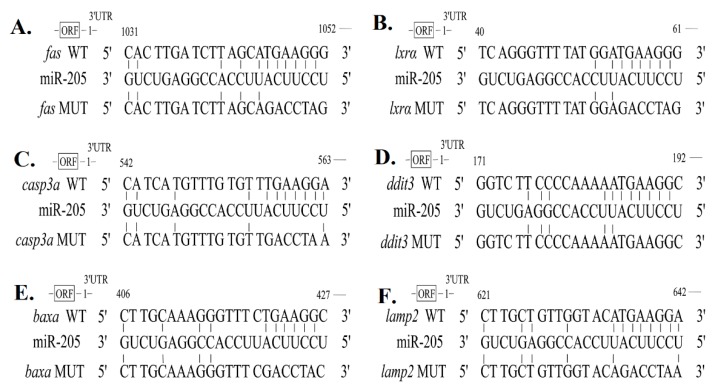
Schematic representation of the miR-205 target sequence within the 3′UTR of *fas*, *lxrα*, *ddit3*, *casp3a*, *baxa* and *lamp2*. (**A**–**F**) The binding sites of miR-205 with *fas*, *lxrα*, *ddit3*, *casp3a*, *baxa*, and *lamp2* 3′UTR. “|” indicates nucleotides that are reversely complementary to miR-205. Six nucleotides (complementary to nucleotides 2–7 of miR-205) were mutated to “GACCTA” in the 3′UTR of *fas*, *lxrα*, *ddit3*, *casp3a*, *baxa* and *lamp2*. The numbers indicate the positions of the nucleotides in the reference wild-type sequences.

To identify the genes through which miR-205 exerts its effects on lipid metabolism, we first used Targetscan Fish 6.2 to predict target genes related to lipid metabolism and the results were compared with the database of hepatic transcriptome of yellow catfish. The alignment of miR-205 with their 3′UTR is illustrated in Figure 4, which indicated that there were predicted miR-205 binding sites in the 3′UTR of *fas*, *lxrα*, *ddit3*, *casp3a*, *baxa* and *lamp2*.

**Figure 5:**
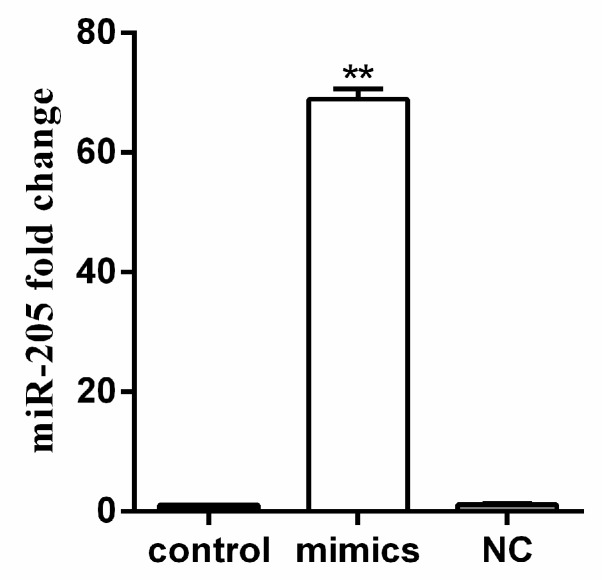
Fold change of miR-205 after miR-205 mimics transfection in HEK-293T at 24 h. miR-205 fold change levels were measured by Q-PCR. miR-205 expression values were normalized to housekeeping genes (*U6*) expressed as a ratio of the control. Mean of the results from the control was set as 1. Data represent means ± SEM (*n* = 3). ** *p* < 0.01, compared to control, independent-samples *T*-test.

**Figure 6:**
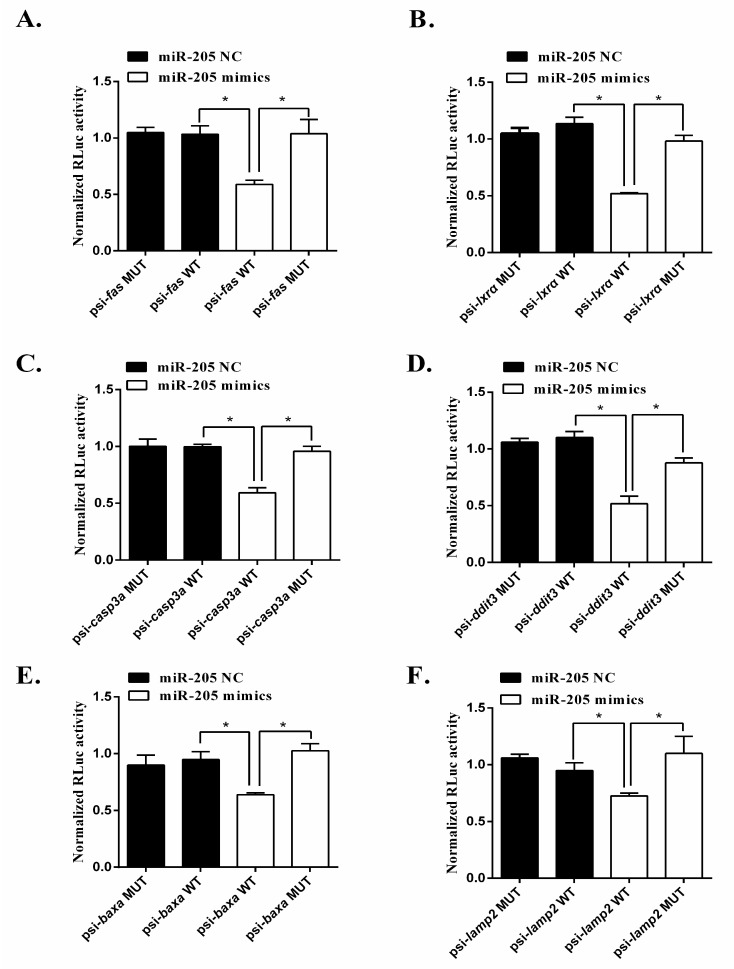
Activities of the luciferase reporter gene linked to the 3′UTR of *fas* (**A**), *lxrα* (**B**), *casp3a* (**C**), *ddit3* (**D**), *baxa* (**E**) and *lamp2* (**F**). The psiCHECK2 luciferase reporter plasmids with the wild-type or mutated 3′UTR sequences of *fas*, *lxrα*, *ddit3*, *casp3a*, *baxa* and *lamp2* were transiently transfected into HEK-29T cells along with 20 μM miR-205 mimics or negative control. Luciferase activities were measured after 24 h, and firefly luciferase was used for normalization. Mean of the results from the cells transfected by psiCHECK2 vector was set as 1. The data are the mean and standard deviation (SD) of separate transfections (*n* = 3). * *p* < 0.05; NS; Student’s *t* test. The experiment was repeated at least three times.

To further validate that they are target genes of miR-205, we acquired the sequences of miR-205 and their 3′UTR from the transcriptomic database of yellow catfish. The luciferase reporter assay was used to verify that the effect of the miR205 is due to direct interaction with the binding sites of the 3′UTRs. As demonstrated in Figure 5, compared to the control, the expression of miR-205 increased about 70 times after the transfection of miR-205 mimics. Cotransfection with miR-205 mimics had a marked repressive effect on wild type RL-*fas*, RL-*lxra*, RL-*casp3a*, RL-*ddit3*, RL-*baxa* and RL-*lamp2*. To further validate the direct interaction between miR-205 and their 3′UTR, we introduced six mutations in the seed sequence of their 3′UTR that is complementary to miR-205. Mutation of the miR-205 sites in *fas*, *lxra*, *casp3a*, *ddit3*, *baxa* and *lamp2* prevented the down-regulation of the reporter activities by miR-205, validating that the effect of the miRNA is due to direct interaction with the binding sites of the 3′UTRs (Figure 6). Taken together, these results strongly suggested that miR-205 could inhibit the expression of *fas*, *lxrα*, *ddit3*, *casp3a*, *baxa*, *lamp2* by binding to the target sites of their 3′UTR.

### 2.4. The Target Gene lxra of miR-205 Mediated Cu-Induced Changes of Lipid Metabolism

**Figure 7:**
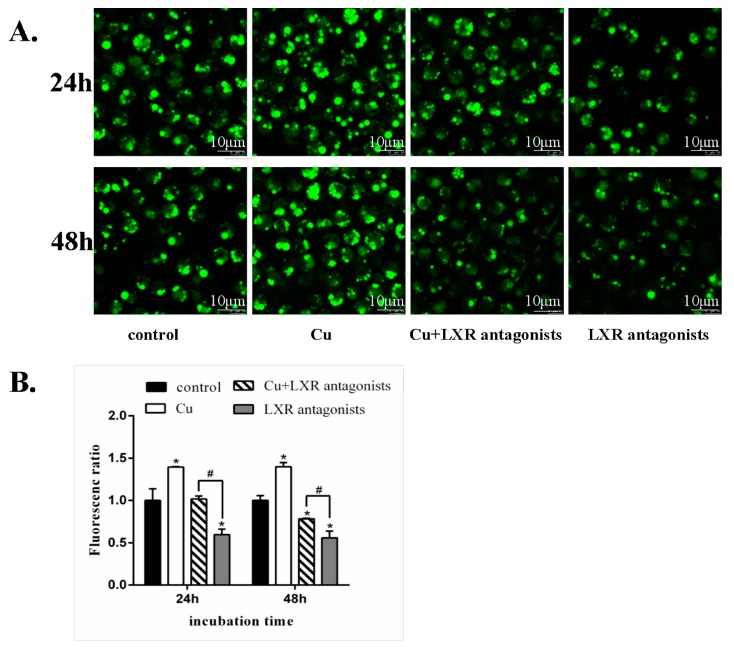
Cu-induced changes in hepatic lipid droplets of yellow catfish. (**A**) Images of hepatocytes stained by BODIPY output by fluorescence scanning confocal microscopy. (**B**) Quantitative analysis of the ratio of fluorescence intensity over area by Image J. Mean of the results from the control was set as 1. The data are mean and standard deviation (SD) of separate transfections (*n* = 3). * *p* < 0.05, compared with control. ^#^ *p* < 0.05, compared with single LXR antagonists incubation (independent-samples *T*-test).

Cu incubation resulted in the increase of the amount and volume of lipid droplets in hepatic cells at 24 and 48 h, based on the intensity of fluorescence after staining by BODIPY (Figure 7A). LXR antagonists reduced lipid droplets in hepatic cells on 24 and 48 h. Compared to the LXR antagonists alone, Cu + LXR antagonists co-incubation increased the amount and volume of lipid droplets in hepatic cells at 24 and 48 h (Figure 7B).

### 2.5. Enzyme Activities and Gene Expression among the Treatments

**Figure 8:**
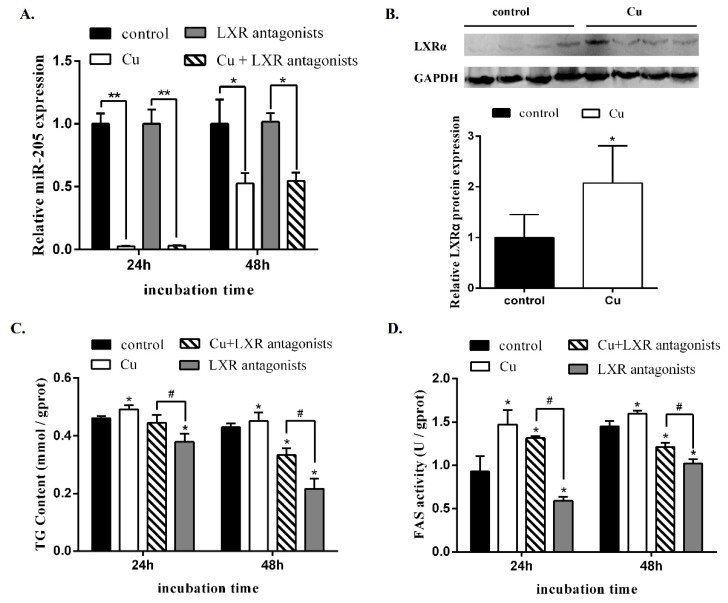
Effects of different treatment for 24 and 48 h on miR-205 expression, LXRα protein expression and FAS activity, and TG content in hepatocytes from yellow catfish. (**A**) Cu exposure inhibited miR-205 expression in vitro at 24 and 48 h. Mean of the results from the control was set as 1. Values are mean ± SEM (*n* = 3). miR-205 expression values were normalized to *U6* expressed as a ratio of the control at 24 and 48h. * *p* < 0.05; ** *p* < 0.01; NS: Student’s *t* test. The experiment was repeated at least three times. (**B**) Cu incubation increased LXRα protein expression. LXRα protein expression level was increased with Cu incubation in vitro at 24 h, based on the image J analysis. Mean of the results from the control was set as 1. Values are mean ± SEM (*n* = 4). LXRα protein expression values were normalized to GAPDH protein expressed as a ratio of the control at 24h. (**C**,**D**) Change of FAS activity and TG content after miR-205 was inhibited by Cu and *lxrα* inhibited by LXR antagonist at 24 and 48h. Mean ± SEM (*n* = 3 independent biological experiments). * *p* < 0.05, compared with control. ^#^ *p* < 0.05, compared with single LXR antagonist incubation (independent-samples *t*-test).

Effects of Cu and LXR antagonist incubation on miR-205 expression at 24 and 48 h were shown in Figure 8A. Compared to the control, Cu incubation down-regulated expression level of miR-205 at both 24 and 48 h, but LXR antagonists did not have significant influence on miR-205 expression at 24 and 48 h. Cu incubation significantly up-regulated LXRα protein expression (Figure 8B).

Compared to the control, Cu incubation significantly increased TG contents and FAS activities at 24 and 48 h. Compared to LXR antagonist, Cu and LXR antagonist coincubation increased TG contents and FAS activities at 24 and 48 h (Figure 8C,D).

Compared to the control, Cu incubation also significantly up-regulated mRNA levels of fas and ddit3 at 24 and 48 h, but did not significantly influence mRNA expression of *lamp2*, *casp3a* and *baxa* (Figure S3).

## 3. Discussion

In recent years, it has become clear that miRNAs play important regulation of gene expression in lipid metabolism and immune response [22]. However, little information is available about molecular mechanisms underlying miRNA-mediated gene regulation of hepatic lipid metabolism in fish. In the present study, we constructed two hepatic small RNA libraries for fish exposed to waterborne Cu and found that miRNA-205 played a potential important role during Cu-induced changes in lipid metabolism by targeting *lxrα*. The results presented here constitute the first study involved in the effects of miRNA-205 mediating Cu-induced changes in lipid metabolism in fish.

In this study, we identified a total of 172 known miRNAs by aligning miRNA sequences with the zebrafish mature miRNAs listed in miRbase 20.0. However, we identified novel miRNAs using MIREAP, most of which were expressed at low levels. These novel miRNAs were added to the known fish miRNA pools. However, the accuracy of these novel miRNA predicted by exploring the secondary structure needs to be further verified. The present study also indicated that 16 miRNAs were differentially expressed between the control and Cu-treated group. Similarly, Wang et al. [23] also found that waterborne Cu exposure influenced miRNAs expression in other fish. Moreover, GO and KEGG analysis indicated that these differentially expressed miRNAs were involved in ether lipid metabolism pathway, glycerophospholipid metabolism pathway and linoleic acid metabolism pathway, which seemed to be important pathways under Cu exposure. Our study also suggests that miR-205 is potential critical regulators of hepatic lipid metabolism of yellow catfish exposed to Cu since most of the target genes of miR-205 are enriched into pathways related to lipid metabolism. Similarly, other studies also suggested the potential roles of miR-205 in lipid metabolism in 3T3-L1 cell lines [24].

It is now well known that miRNAs regulate a variety of cellular pathways through the regulation of expression of multiple target genes. In mammals, miRNAs are believed to bind through partial homologous sequence to a target gene at 3′UTR, causing translational repression. In the present study, searching the complete 3′UTR by manual inspection revealed that *fas*, *lxrα*, *ddit3*, *casp3a*, *baxa* and *lamp2* contain putative sites for miR-205, which implied that miR-205 directly targets *fas*, *lxrα*, *ddit3*, *casp3a*, *baxa* and *lamp2* at the 3′UTR. These data are highly suggestive that miR-205 plays important roles in regulating the function of liver tissues. To validate whether these predicted target genes are targeted by miRNA-205, we attached their 3′UTRs to Renilla luciferase (RL) reporter genes and compared reporter activities. Reporter assays revealed that these targets were uniformly repressed by miR-205. As critical genes for lipid synthesis, main function of FAS is to catalyze the synthesis of palmitate from acetyl-CoA and malonyl-CoA, in the presence of NADPH, into long-chain saturated fatty acids [25]. LXR was an obligatory intermediary component in regulation of lipid metabolism by insulin [26]. *ddit3* is multifunctional transcription factor in ER stress response [27], and ER stress has effect of lipid metabolism [20]. *lamp2* plays an important role in chaperone-mediated autophagy, a process that mediates lysosomal degradation of proteins in response to various stresses [28], and autophagy regulates lipid metabolism [29,30]. *casp3a* and *baxa* were key genes in the process of apoptosis [31], and apoptosis has been shown to be associated with lipid metabolism [32,33]. Thus, identification of *fas*, *lxrα*, *ddit3*, *casp3a*, *baxa* and *lamp2* as mir-205 target gene provides a possible mechanism for Cu-induced changes of lipid metabolism. 

In the present study, although Cu down-regulated expression levels of miR-205, and *lxrα*, *casp3a*, *baxa*, and *lamp2* are putative target genes of miR-205, Cu did not influence mRNA levels of *lxrα*, *casp3a*, *baxa* and *lamp2*. These two observations are not necessarily contradictory. It might account for the observation that many predicted miRNA targets do not seem to be regulated by a given miRNA [34,35]. These results also highlight the importance of in vivo validation of putative targets by testing the role of their full-length 3′UTRs. 

The present study indicated that Cu incubation increased the TG contents of the hepatocytes, in agreement with our previous studies [20]. Moreover, compare to the LXR antagonists alone, Cu + LXR antagonists co-incubation increased TG contents and FAS activity, indicating that LXRa mediated Cu-induced changes in lipid metabolism. The present study also indicated that compared to the control, Cu incubation down-regulated expression level of miR-205 at both 24 and 48 h, similar to the in vivo studies. Cu incubation significantly up-regulated LXRα protein expression but did not significantly influence *lxra* mRNA expression, indicating that Cu influenced LXRa expression at the translational levels. Therefore, our data showed that miR-205 down-regulates LXR expression, suggesting that LXRa is the major target through which miR-205 mediates its effects on Cu-induced changes of lipid metabolism.

In conclusion, we have characterized a large number of miRNAs in yellow catfish, including six novel miRNAs not previously described in other species, which will be useful for further studies in this commercially important species. The target mRNAs of miR-205 were predicted and validated, and interesting targets were found which should be explored in the future. We provide evidence that miR-205 mediated the Cu-induced changes of lipid metabolism by targeting *lxra*.

## 4. Material and Methods

### 4.1. Ethics Statement

This study followed protocols for the care and use of laboratory animals established by the Institutional Animal Care and Use Committee (IACUC) at Huazhong Agricultural University (Wuhan, China). All yellow catfish surgery was performed on ice to decrease suffering.

### 4.2. Chemical Reagents

Cu was added as CuSO_4_·5H_2_O (AR, Shanghai Sinopharm Group Corporation, Shanghai, China) and the stock solution was prepared with sterile double-distilled H_2_O to a concentration of 0.1 M. MS-222 and l-glutamine were obtained from Amresco (Solon, OH, USA). Medium 199 (M199), DMEM (high glucose) and fetal bovine serum (FBS) were obtained from Gibco/Invitrogen (Paisley, UK). Penicillin and streptomycin were obtained from Sigma-Aldrich (St. Louis, MO, USA). miR-205 mimics and miR-205 negative control were purchased from Shanghai GenePharma Co., Ltd. (Shanghai, China). LXR Antagonists (SR9243) was purchased from MedChemexpress (Monmouth Junction, NJ, USA). BODIPY (D3922, Molecular Probes, Carlsbad, CA, USA).

### 4.3. Experimental Treatments

Three experiments were conducted. In Expt. 1, *P. fulvidraco* were exposed to waterborne Cu for 60 days and miRNAs which mediated the Cu-induced changes of lipid metabolism were screened. In Expt. 2, luciferase reporter gene was used to verify the target genes of miRNA-205. In Expt. 3, primary hepatocytes of *P. fulvidraco* were used to examine the effects of the target gene *lxra* on miR-205 mediating Cu-induced changes in hepatic lipid metabolism.

#### 4.3.1. Experiment 1: In Vivo Study: Investigating the Effects of Waterborne Cu Exposure on Hepatic miRNAs in Yellow Catfish

Juvenile yellow catfish were obtained from a local pond and transferred to indoor cylindrical fiberglass tanks (300 L in water volume). After, 60 uniform-sized fish (initial mean weight: 15.2 ± 0.2 g, mean ± SEM) were randomly assigned to 6 fiberglass tanks with 10 fish per tank. They were exposed to two nominal Cu concentrations of zero (control) and 60 μg/L, respectively, with triplicates for each concentration. Cu concentration was selected, based on our recent study [20]. Cu concentrations in the test tanks were monitored twice every week by ICP-MS measurements [20], and the measured values for two treatments were 1 ± 1 μg Cu/L and 62 ± 5 μg Cu/L, respectively. During the exposure period, half of the water in each tank was replaced daily with fresh dechlorinated water containing the corresponding Cu concentration. The fish were fed twice daily with a commercial pellet diet (Haida^®^, Wuhan, China) at a rate of 3% of body weight. The amount of feed consumed by the fish in each tank was recorded daily, and the results showed that Cu exposure did not affect the feeding rate (data not shown). No mortality was observed during the experimental period. The experiment continued for 60 days. During the experiment, fish was subjected to ambient environment and normal photoperiod at 14L: 10D, and water temperature, 22.7–26.8 °C; pH, 7.8–7.9; dissolved oxygen, 6.4–7.2 mg/L; total hardness, 143.5–153.7 mg/L as CaCO_3_. These parameters of water quality showed no significant differences among the test tanks.

At the end of the 60-day period, the fish were starved for 24h before sampling. Yellow catfish were euthanized with MS-222 (tricaine methanesulfonate, 0.38 mM), and liver tissues were quickly taken from each individual. Total RNA was isolated from each sample using TRIzol Reagent (Invitrogen, Carlsbad, CA, USA) based on the guanidinium thiocyante-phenol-chloroform extraction method. The potential DNA contamination were eliminated by the addition of RNase-Free DNase. RNA from the same tissues was combined into one RNA pool, whose quality and quantity was measured with NanoDrop 2000 and Agilent 2100 Bioanalyzer, quality control (OD_260_/OD_280_ > 1.8 and OD_260_/OD_230_ > 1.5). Finally, RNA samples were sent to Beijing BerryGenomics Company (Beijing, China) to establish miRNA libraries and sequenced using an Illumina/Solexa Genome Analyzer (Illumina, Beijing, China).

#### 4.3.2. Experiment 2: The Verification of Target Genes of miR-205

Human embryonic kidney (HEK) 293T cells were maintained in Dulbecco′s modified Eagle′s medium (DMEM) with 10% FBS, 100 U/mL penicillin, 100 mg/mL streptomycin, and 250 ng/mL amphotericin B (Invitrogen) and incubated at 5% CO_2_ at 37 °C. 400–600 bp wild type DNA fragments of the 3′UTRs of *fas*, *lxrα*, *ddit3*, *casp3a*, *baxa* and *lamp2* mRNA containing the putative binding sites of miR-205 were synthesized by PCR. The fragments were then subcloned into the *XhoI* and *NotI* sites downstream of the Renilla luciferase coding region in the psiCHECK-2 vector (Promega, Madison, WI, USA), and ligated using ClonExpress™ II One Step Cloning Kit (Vazyme, Piscataway, NJ, USA). Mutant recombinant plasmid was only mutates 6mer which was complementary to miR-205 seed sequence to GACCTA by overlap-PCR, validated by sequencing by Tsingke (Wuhan, China). The PCR reactions were performed using TaKaRa PrimeSTAR^®^ HS DNA Polymerase kit as mentioned above. HEK-293T cells were plated at 1 × 10^5^ cells/well on 24-well plates and co-transfected with psiCHECK2-*fas*-3′UTR (psi-*fas*) (0.2 μg), psiCHECK2-mut*fas*-3′UTR (psi-*fas*-mut) (0.2 μg), respectively, or empty vector plasmid (0.2 μg) and miR-205 mimics (20 pmol), miR-205 negative control (20 pmol) using Lipofectamine2000 (Invitrogen) (2 μL). Cells were harvested at 48h transfection.

#### 4.3.3. Experiment 3: Investigating the Potential Role of *lxra* (miR-205 target gene) Mediating the Cu-Induced Changes of Lipid Metabolism

Hepatocytes were isolated from *P. fulvidraco* (mean weight: 10.4 ± 0.4 g, mean ± SEM) liver according to our published protocols [36,37]. For the experiment, four groups were designed as follows: Control, LXR antagonists (10 µM), Cu (10 µM), LXR antagonists (10 µM) + Cu (10 µM). Each treatment was performed in triplicate. LXR antagonists was added 2 h prior to the addition of Cu. The cells were gathered for the following analysis after 24 and 48 h.

### 4.4. Sample Analysis

#### 4.4.1. Small RNA Library Sequencing Analysis

Raw reads obtained from Solexa sequencing were processed to obtain clean reads by summarizing data production and evaluating sequencing quality. After removing adapter sequences and low-quality reads, high-quality reads between 16 and 30 nt in length were processed for bioinformatics analysis with a proprietary software package. The sequencing sequences were searched against pre-miRNA (pre-miR) and mature miRNA (miR) sequences from selected species (*Deuterostoma*) listed in the miRBase v20.0 (http://www.mirbase.org/). Reads mapped to mRNA in NCBI GenBank, non-coding RNAs (rRNA, tRNA, snoRNA, snRNA and others) in Rfam (http://rfam.janelia.org) and repetitive sequence elements in RepBase (http://www.girinst.org/repbase) were removed before further analysis. The retain reads were aligned to *Danio rerio* genome and EST sequences as primary source of reference since the *P. fulvidraco* genome was not available [38]. Flanking sequences from each mapping locus were subjected to secondary structure analysis using RNAfold (http://rna.tbi.univie.ac.at/cgi-bin/RNAfold.cgi) with the default folding criteria. The nohit-reads were blasted against the piRNA database download from piRNA cluster-database.

In order to obtain miRNAs in Cu-treated group, each of identified miRNA read was normalized to the total number of miRNA reads in each library and multiplied by a million. If the normalized expression (NE) of a certain miRNA was lower than 1 in each group, further differential expression analysis was conducted without this miRNA. Results of the Audic-Claverie test, Fisher exact test, and Chi-squared 262 test with a Bonferroni correction for multiple comparisons and a *p*-value < 0.05 indicated a unique differentially expressed miRNA. After normalized miRNA read count, the |log_2_fold-change| and *p*-value were calculated from the NE data and the statistical significance of miRNA expression was compared between the control and Cu-treated group. Among two groups, when *p*-value < 0.05, and |log_2_foldchange| > 1.0 or <1.0, a specific miRNA was designated as up-regulation or down-regulation. Scatter plots were used to demonstrate differentially expressed miRNA between two groups.

#### 4.4.2. The Prediction of Target Genes for miRNAs

The target genes of differentially expressed miRNAs were predicted by TargetScanFish 6.2 (http://www.targetscan.org/fish_62/). Because the zebrafish genome does not completely match the genome of the yellow catfish, we compared the predicted results with the hepatic transcriptome of the yellow catfish from our laboratory. The screening was based on the principle of Targetscan target gene prediction, as follows: targets of miRNAs were searched in 3′UTR of mRNA for the presence of conserved 8mer (an exact match to positions 2–8 of the mature miRNA (the seed + position 8) followed by an ‘A’), 7mer (7mer-m8: An exact match to positions 2–8 of the mature miRNA (the seed + position 8); 7mer-A1: An exact match to positions 2–7 of the mature miRNA (the seed) followed by an ‘A’), and 6mer (An exact match to positions 2–7 of the mature miRNA (the seed)) sites that match the “seed region” of each miRNA [39]. In order to explore the potential pathways regulated by these miRNAs, the target genes of the differentially expressed miRNAs were subjected to the analysis of Gene Ontology (GO) (http://www.geneontology.org/) and KEGG pathway (http://www.genome.jp/kegg/). The Bonferroni Correction was carried out to correct *p*-values, only GO terms with *p* ≤ 0.05 and pathways with FDR ≤ 0.05 were identified as highly represented.

#### 4.4.3. Luciferase Reporter Gene Assay Analysis

Firefly and renilla luciferase activities were analyzed using the Dual-Luciferase Report assay (Promega) according to the manufacturer′s instructions. Luciferase activity values were obtained using a Turner Biosystems 20/20n single-tube luminometer. Renilla luciferase activity was first normalized to the firefly luciferase activity, and then this ratio was normalized to the control constructs noted for each experiment. At least four independent transfections for each condition were averaged. All experiments were performed in triplicates and measured at least three times.

#### 4.4.4. miRNA Expression Level Determination by Real-Time Q-PCR

The total RNAs were extracted using TRIzol regent (Takara, Tokyo, Japan) according to manufacturer′s recommendations. mRNA reverse transcriptions were performed with equal quantities of each total RNA (1 μg) as template using Quantitect Reverse Transcription Kit (Takara) for real-time PCR following the manufacturer′s protocol. Real-time quantification of microRNAs was performed by stem-loop RT-PCR [36], miR-205 stem-loop RT primers (5′-CTCAACTGGTGTCGTGGAGTCGGCAATTCAGTTGAGCAGACTCC-3′) replaced Oligo dT/Random primers in TAKARA Reverse Transcription Kit. The resulting first-strand cDNA was diluted to 1:10 with ddH_2_O and used as template for SYBR real-time PCRs.

Real-time Q-PCR was performed using the SYBR Premix Ex TaqTM II kit (Takara) on a Chromo 4 Real-Time Detection System (MJ Research, Hercules, CA, USA) following the protocols in our recent publication [20]. The gene-specific primers for each gene are listed in Table S1. miRNA expression of each gene was determined respectively by comparative delta-delta Ct method normalized with *β-actin* and *U6* [40]. β-actin and tuba showed the most stable level of expression under the in vitro experiment and accordingly were chosen as the housekeeping genes. To confirm amplification specificity, the PCR products from each sample were examined by melting curve analysis and subsequent agarose gel electrophoresis. All experiments were performed in triplicates.

#### 4.4.5. Western Blot

Western blot was used to determine the protein expression of LXR following Wei et al. [29]. Briefly, cell lysates were prepared with RIPA buffer (Invitrogen, Carlsbad, CA, USA). 60 μg protein were separated on a 12.5% SDS-PAGE gels. After electrophoresis, proteins were transferred to a PVDF membrane, and then blocked with 8% (*w*/*v*) dry milk for 2 h. The membrane was incubated with antibodies against N1RH3 (Proteintech Group, Wuhan, China) overnight at 4 °C, and then processed with goat anti-rabbit IRDye 800CW secondary antibody (926-32211, Li-Cor Biosciences). The protein bands were visualized by Odyssey Infrared Fluorescent Western Blots Imaging200 System from Li-COR Bioscience (Lincoln, NE, USA). Stripping Buffer (CWBIO, Beijing, China) was used to wash membrane, which was then blocked with 8% (*w*/*v*) dry milk for 2 h. The membrane was incubated with antibodies against GAPDH (Abcam, Cambridge, UK) 1.5h at room temperature, and then processed with goat anti-rabbit IRDye 800CW secondary antibody (926-32211, Li-Cor Biosciences). The protein bands were visualized by Odyssey Infrared Fluorescent Western Blots Imaging200 System from Li-COR Bioscience, and quantified by Image-Pro Plus (Media Cybernetics, Sliver Spring, MD, USA).

#### 4.4.6. Analysis of TG Content and FAS Activity

TG was determined by glycerol-3-phosphate oxidase p-aminophenol (GPO-PAP) methods, using a commercial kit from Nanjing Jiancheng Bioengineering Institute, Nanjing, China. The cellular TG content was expressed as mmol TG per gram cellular protein. FAS activity was determined by using a commercial kit from Nanjing Jiancheng Bioengineering Institute, Nanjing, China, and the relevant protocols were described in our recent publication [20]. Enzyme activity (IU) was expressed as units (IU) per gram of soluble protein. Soluble protein concentration of homogenates was determined by the method of Song et al. [20], using bovine serum albumin (BSA) as standard. These analyses were conducted in triplicate.

#### 4.4.7. Intracellular Lipid Droplets Staining

Cells were washed three times with PBS and incubated with 5 μg/mL BODIPY (D3922, Molecular Probes, Carlsbad, CA, USA) (excitation wavelength 480 nm, emission maximum 515 nm) for 30 min. Cell then were washed three times with PBS, and hepatocytes were observed using the laser scanning confocal microscope (Leica, Bensheim, Germany) to visualize the intensity of fluorescence.

### 4.5. Statistical Analysis

Statistical analysis was performed with SPSS 19.0 software. Results were presented as mean ± standard error of means (SEM). Prior to statistical analysis, an arcsine transformation was used before processing percentage data. For the same Cu and LXR antagonists concentrations, different group were analyzed by Student′s T-test for independent samples. The minimum significant level was set at 0.05.

## Supplementary Materials

Supplementary materials can be found at http://www.mdpi.com/1422-0067/19/10/2980/s1.
