# Supplementary material for: MiR-205 Mediated Cu-Induced Lipid Accumulation in Yellow Catfish Pelteobagrus fulvidraco
Source: Int J Mol Sci. 2018 Sep 29;19(10):2980. doi: 10.3390/ijms19102980 (PMC6213235; doi:10.3390/ijms19102980)
Supplement: Supplementary file 1 [file ijms-19-02980-s001.pdf]

Supplementary Table 1 Primers used for real-time quantitative PCR analysis.

Genes	Forward primer (5'-3')	Reverse primer (5'-3')	Size	Accession no.
fas	AACTAAAGGCTGCTGGTTGCTA	CACCTTCCCGTCACAAACCTC	141	MH267731
ddit3	ACTGTCCCAAACTCATCCCC	ACTTCCTTCCCGTAACCCCT	349	MG685920
casp3a	CTCATTTGTTTGCGTGTTGC	CACTGGGATTTTCATCGAATTATCT	223	KY072821
baxa	TCGGAGACGAACTGGACAAC	TCGACAAGCAAAGTAGAAAAGC	167	KY072819
lamp2	CTGAGGGCAAGGATGGGTTT	TTGGTGAAGACAAACTGGACAA	217	MH357355
miR-205	TCGGCAGGTCCTTCATTCCACC	CTCAACTGGTGTCGTGGA	58	MI0001378
β-actin	GTGCGTGACATCAAGGAGAAG	CGAGGAAGGATGGCTGGAA	178	KP893561
U6	CTCGCTTCGGCAGCACA	AACGCTTCACGAATTTGCGT	134	NM_00103460
tuba	CACTTCCCTCTTGCCACCTA	ACGGTACAGGAGACAACAGG	165	KP893558

Supplementary Table 2: miRNA information in control and Cu-treated group.

Mature-miRNA	Mature-miRNA-seq	T_Rds-Exp	C1_Rds-Exp	T_Rds-Exp-TPM	C1_Rds-Exp-TPM	Fold-change	p-value
dre-miR-122	TGGAGTGTGACAATGGTGTTTG	2054573	2038507	170403.0991	164897.1728	-0.04738491	0.892615007
dre-let-7a	TGAGGTAGTAGGTTGTATAGTT	496228	712182	41156.38094	57609.22004	0.4851836	0.614421936
dre-miR-21	TAGCTTATCAGACTGGTGTTGGC	440213	373517	36510.58369	30214.21918	-0.273087074	0.685007001
dre-miR-199-5p	CCCAGTGTTCAGACTACCTGTTC	281809	246665	23372.80153	19953.01519	-0.228223895	0.724905934
dre-miR-22a	AAGCTGCCAGCTGAAGAACTGT	223738	287589	18556.48283	23263.40455	0.326138958	0.753976754
dre-miR-30e-5p	TGTAAACATCCTTGACTGGAAG	206292	207191	17109.53863	16759.91798	-0.029785769	0.90935227
dre-miR-101a	TACAGTACTGTGATAACTGAAG	192335	241799	15951.96669	19559.39886	0.294127728	0.783302162
dre-miR-26b	TTCAAGTAATCCAGGATAGGTT	183498	213780	15219.03962	17292.90976	0.18430332	0.886106688
dre-let-7e	TGAGGTAGTAGATTGAATAGTT	139119	196131	11538.31416	15865.26188	0.459438885	0.636223508
dre-miR-148	TCAGTGCATTACAGAACTTTGT	136302	138972	11304.67655	11241.60471	-0.008071723	0.930054602
dre-miR-101b	TACAGTACTATGATAACTGAAG	127890	155346	10606.99831	12566.11638	0.244522403	0.829372572
dre-miR-26a	TTCAAGTAATCCAGGATAGGCT	127431	154165	10568.92956	12470.58393	0.238699755	0.834823506
dre-miR-142a-3p	TGTAGTGTTTCCTACTTTATGGA	106838	120616	8860.978073	9756.766787	0.138937192	0.929278818
dre-miR-146a	TGAGAACTGAATTCCATAGATGG	105908	102120	8783.845315	8260.604102	-0.088605358	0.853654103
dre-let-7b	TGAGGTAGTAGGTTGTGTGGTT	97905	136273	8120.088903	11023.2795	0.440986071	0.652054221
dre-miR-144	TACAGTATAGATGATGTACT	95555	100222	7925.183547	8107.072701	0.032736732	0.96908045
dre-miR-126a-3p	TCGTACCGTGAGTAATAATGC	92037	77786	7633.406081	6292.19889	-0.278762668	0.680042425
dre-miR-19a-3p	TGTGCAAATCTATGCAAAACTGA	90528	74672	7508.251961	6040.303853	-0.31385594	0.649547024
dre-miR-29a	TAGCACCATTTGAAATCGGTTA	82441	95812	6837.52872	7750.342736	0.180785123	0.889454217
dre-miR-19b-3p	TGTGCAAATCCATGCAAAACTGA	81419	62041	6752.765624	5018.567754	-0.428202792	0.554551144
dre-miR-192	ATGACCTATGAATTGACAGCC	79200	89480	6568.725205	7238.139982	0.140005596	0.928264183
dre-miR-125b	TCCCTGAGACCCTAACTTGTGA	74354	84947	6166.805478	6871.460406	0.156093411	0.912928328
dre-miR-451	AAACCGTTACCATTACTGAGTT	72938	74864	6049.364633	6055.834954	0.001542263	0.939245315
dre-miR-181a-5p	AACATTCAACGCTGTCGGTGAGT	70680	66042	5862.089614	5342.21324	-0.133977459	0.8111912
dre-miR-30d	TGTAAACATCCCCGACTGGAAG	67980	70124	5638.155801	5672.410909	0.0087387	0.946124476
dre-miR-100	AACCCGTAGATCCGAACTTGTG	60237	68278	4995.963386	5523.085848	0.144711648	0.923780675
dre-miR-25	CATTGCACTTGTCTCGGTCTGA	59423	67983	4928.451488	5499.22296	0.158093355	0.911029219
dre-miR-194a	TGTAACAGCAACTCCATGTGG	51768	53372	4293.557656	4317.322386	0.007963262	0.945390206
dre-let-7g	TGAGGTAGTAGTTTGTATAGTT	46141	63603	3826.863001	5144.919728	0.426986314	0.66419272

dre-miR-19d	TGTGCAAACCCATGCAAAACTGA	37717	33182	3128.189502	2684.130095	-0.220873314	0.731573229
dre-miR-128	TCACAGTGAACCGGTCTCTTTT	36678	43638	3042.016453	3529.927945	0.214610779	0.857491939
dre-miR-457a	AAGCAGCACATCAATATTGGCA	31795	27485	2637.028004	2223.293221	-0.24621466	0.708871529
dre-miR-143	TGAGATGAAGCACTGTAGCTC	31764	25484	2634.456912	2061.430033	-0.353860088	0.615570497
dre-miR-2188-5p	AAGGTCCAACCTCACATGTCC	31021	32152	2572.833644	2600.812212	0.015604058	0.952715844
dre-let-7f	TGAGGTAGTAGATTGTATAGTT	29840	36319	2474.883335	2937.885628	0.247417714	0.826709842
dre-miR-152	TCAGTGCATGACAGAACTTTGG	28452	27992	2359.764767	2264.305033	-0.059574729	0.881116663
dre-miR-462	TAACGGAACCCATAATGCAGCT	27560	30658	2285.78367	2479.960836	0.117628466	0.94967688
dre-miR-93	AAAAGTGCTGTTTGTGCAGGTA	27492	26390	2280.143855	2134.717413	-0.095079744	0.847613778
dre-miR-92a	TATTGCACTTGTCCCGGCCTGT	26298	30576	2181.115346	2473.327762	0.181387368	0.888924596
dre-miR-16c	TAGCAGCATGTAAATATTGGAG	26187	28056	2171.909178	2269.482067	0.063399312	0.998492992
dre-miR-223	TGTCAGTTTGTCAAATACCCC	25404	17373	2106.968372	1405.321926	-0.584268003	0.437471435
dre-miR-16b	TAGCAGCACGTAAATATTGGAG	24656	27791	2044.930412	2248.045913	0.136619751	0.93153808
dre-miR-130c	CAGTGCAATATTAAAAGGGCAT	24531	23812	2034.563106	1926.180032	-0.078976477	0.862786367
dre-miR-222a	AGCTACATCTGGCTACTGGGTCTC	23737	30043	1968.709977	2430.212779	0.303832041	0.774443645
dre-miR-221	AGCTACATTGTCTGCTGGGTTTC	21911	48882	1817.264368	3954.121129	1.121588755	0.209345452
dre-miR-199-3p	TACAGTAGTCTGCACATTGGTT	20911	19321	1734.325919	1562.897883	-0.15015152	0.796251996
dre-miR-190a	TGATATGTTTGATATATTAGGT	20656	20784	1713.176614	1681.241634	-0.0271468	0.91193639
dre-miR-181b	AACATTCATTGCTGTCGGTGGG	19707	17671	1634.468025	1429.427488	-0.193383717	0.756544328
dre-let-7j	TGAGGTAGTTGTTTGTACAGTT	18813	20133	1560.321051	1628.581496	0.061773007	0.996954708
dre-miR-99	AACCCGTAGATCCGATCTTGTG	18299	17927	1517.690688	1450.135622	-0.065689961	0.875362904
dre-miR-103	AGCAGCATTGTACAGGGCTATGA	18093	15529	1500.605368	1256.158648	-0.256525942	0.699761475
dre-miR-20a-5p	TAAAGTGCTTATAGTGCAGGTAG	18080	17263	1499.527168	1396.423899	-0.102770707	0.840427433
dre-miR-722	TTTTTTGCAGAAACGTTTCAGATT	17343	24073	1438.401531	1947.292622	0.437003235	0.655584547
dre-miR-454b	TAGTGCAATATTGCTTATAGGG	16712	17673	1386.067369	1429.58927	0.044603331	0.98051508
dre-miR-126b-5p	CATTATTACTTTTGGTACGCG	16338	17931	1355.048389	1450.459187	0.09816533	0.968336606
dre-miR-126a-5p	CATTATTACTTTTGGTACGCG	16338	17931	1355.048389	1450.459187	0.09816533	0.968336606
dre-let-7h	TGAGGTAGTAAGTTGTGTTGTT	14442	19279	1197.797088	1559.500455	0.380700443	0.704980874
dre-miR-200a	TAACACTGTCTGGTAACGATGT	12675	25765	1051.244848	2084.160445	0.987367615	0.271989704
dre-miR-210-3p	CTGTGCGTGTGACAGCGGCTAA	12124	23111	1005.545762	1869.475337	0.894654698	0.322387716
dre-miR-30c	TGTAAACATCCTACACTCTCAG	11406	10919	945.9959556	883.2504523	-0.099011433	0.84401683

dre-miR-130b	CAGTGCAATAATGAAAGGGCAT	11004	10309	912.6546989	833.9068516	-0.130182881	0.814875792
dre-let-7c	TGAGGTAGTAGGTTGTATGGTT	9599	12201	796.1261772	986.9529048	0.309984144	0.768914891
dre-miR-456	CAGGCTGGTTAGATGGTTGTCA	9318	7675	772.8204729	620.839566	-0.31591281	0.648021498
dre-miR-30e-3p	CTTTCAGTCGGATGTTTGCAGC	9218	10253	764.526628	829.3769473	0.117461197	0.94994298
dre-miR-203a	GTGAAATGTTTAGGACCACTTG	7249	33640	601.2208208	2721.178241	2.178264581	0.014760126
dre-miR-27b	TTCACAGTGGCTAAGTTCTGCA	7225	7170	599.230298	579.9895359	-0.047083699	0.893137311
dre-miR-146b	TGAGAACTGAATTCCAAGGGTG	7179	7901	595.4151293	639.1209656	0.102193134	0.964592795
dre-miR-15b	TAGCAGCACATCATGGTTTGTA	7082	6080	587.3700997	491.8181839	-0.25614475	0.700303396
dre-miR-145	GTCCAGTTTTCCCAGGAATCCC	5472	4514	453.8391959	365.1426451	-0.313721042	0.65009673
dre-miR-10c	TACCCTGTAGATCCGGATTTGT	5441	5288	451.268104	427.7523941	-0.077208882	0.864713845
dre-miR-125c	TCCCTGAGACCCTAACTCGTGA	5142	5252	426.4695076	424.8403128	-0.005521924	0.932782577
dre-miR-135c	TATGGCTTTCTATTCCTATGTG	4957	5651	411.1258944	457.1158811	0.1529797	0.916186124
dre-miR-29b	TAGCACCATTTGAAATCAGTGT	4820	4677	399.7633268	378.3279023	-0.079508948	0.862587934
dre-miR-150	TCTCCCAATCCTTGTACCAGTG	4776	5743	396.1140351	464.5578668	0.229942495	0.843380322
dre-miR-140-3p	TACCACAGGGTAGAACCACGGAC	4759	4156	394.7040814	336.1836138	-0.231522031	0.722420306
dre-miR-301a	CAGTGCAATAGTATTGTCAAAG	3753	3781	311.2680012	305.8494331	-0.025335671	0.914018149
dre-miR-338	TCCAGCATCAGTGATTTTGTTG	3713	3298	307.9504632	266.7790083	-0.207053147	0.744607075
dre-miR-19c	TGTGCAAATCCATGCAAAACTCG	3475	3318	288.2111122	268.3968313	-0.102758322	0.840880638
dre-miR-205	TCCTTCATTCCACCGGAGTCTG	3224	14888	267.3935614	1204.307421	2.171167081	0.015100748
dre-miR-22b	AAGCTGCCAGTTGAAGAGCTGT	3172	3808	263.080762	308.0334941	0.227581477	0.84577668
dre-miR-130a	CAGTGCAATGTTAAAAGGGCAT	2949	2903	244.5854877	234.8270046	-0.058740481	0.882439364
dre-miR-15a-5p	TAGCAGCACAGAATGGTTTGTG	2862	2728	237.3698426	220.6710536	-0.105239258	0.838673339
dre-miR-107b	AGCAGCATTGTACAGGGCTTT	2804	2720	232.5594125	220.0239244	-0.079938928	0.862454415
dre-let-7i	TGAGGTAGTAGTTTGTGCTGTT	2768	3006	229.5736284	243.1587929	0.082941836	0.983331961
dre-miR-222b	AGCTACATCTGAATACTGGGTCA	2712	2750	224.9290752	222.4506588	-0.01598479	0.92308559
dre-miR-1388-3p	ATCTCAGGTTCGTCAGCCCATG	2651	4051	219.8698298	327.6900432	0.575682185	0.541430813
dre-miR-107a	AGCAGCATTGTACAGGGCTATCA	2649	2817	219.7039529	227.8703658	0.05265249	0.988660145
dre-miR-200b	TAATACTGCCTGGTAATGATGA	2539	5372	210.5807234	434.5472506	1.04513968	0.244046675
dre-miR-181c	CACATTCATTGCTGTCGGTGGG	2522	2532	209.1707698	204.8163884	-0.030350101	0.909463344
dre-miR-301c	CAGTGCAATAGTATTGTCATAG	2463	2283	204.2774012	184.6744924	-0.145544999	0.801248311
dre-miR-7b	TGGAAGACTTGTGATTTTGTT	2450	2022	203.1992014	163.5619026	-0.313057982	0.651235352

dre-miR-16a	TAGCAGCACGTAAATATTGGTG	2386	2553	197.8911406	206.5151025	0.061540264	0.997221055
dre-miR-1	TGGAATGTAAAGAAGTATGTAT	1957	1567	162.3105458	126.75643	-0.356697806	0.614446762
dre-miR-17a-5p	CAAAGTGCTTACAGTGCAGGTA	1924	1656	159.5735769	133.9557422	-0.252465357	0.704461397
dre-miR-23a	ATCACATTGCCAGGGATTTCCA	1769	1306	146.7181173	105.6438402	-0.473838381	0.520284995
dre-miR-27c-3p	TTCACAGTGGTTAAGTTCTGC	1720	1990	142.6541332	160.9733858	0.174300636	0.896516541
dre-miR-23b	ATCACATTGCCAGGGATTACCA	1697	1440	140.7465489	116.4832541	-0.272976983	0.686521712
dre-miR-301b	CAGTGCAATAGTATTGTCATTG	1682	1544	139.5024721	124.8959335	-0.159564183	0.788692045
dre-miR-24	TGGCTCAGTTCAGCAGGAACAG	1650	1539	136.8484418	124.4914778	-0.136532023	0.810004294
dre-miR-92b	TATTGCACTCGTCCCGGCCTCC	1650	2113	136.8484418	170.9229971	0.320767513	0.759980931
dre-miR-30a	TGTAAACATTCCCGACTGGAAG	1618	1688	134.1944114	136.5442589	0.025044066	0.962604707
dre-miR-455a	TATGTGCCCTTGGACTACATCG	1556	1081	129.0522275	87.44333171	-0.561534768	0.455349377
dre-miR-210-5p	AGCCACTGACTAACGCACATTG	1451	2627	120.3436903	212.5010475	0.820309451	0.368227195
dre-miR-126b-3p	TCGTACCGTGAGTAATAGTGCA	1409	1073	116.8602754	86.79620252	-0.429080766	0.555784482
dre-miR-217	TACTGCATCAGGAACTGATTGG	1376	1948	114.1233066	157.5759576	0.465453977	0.632519601
dre-let-7d	TGAGGTAGTTGGTTGTATGGTT	1315	2527	109.0640612	204.4119327	0.906303635	0.31689091
dre-miR-140-5p	CAGTGGTTTTACCCTATGGTAG	1291	1171	107.0735384	94.72353509	-0.176807155	0.773243749
dre-miR-124	TAAGGCACGCGGTGAATGCCAA	1119	1203	92.80812505	97.31205184	0.068367376	0.998001152
dre-miR-458	ATAGCTCTTTGAATGGTACTGC	1051	1226	87.16831048	99.17254826	0.186137079	0.885896749
dre-miR-27a	TTCACAGTGGCTAAGTTCCGCT	1048	1074	86.91949513	86.87709367	-0.000703954	0.938557431
dre-miR-429a	TAATACTGTCTGGTAATGCCGT	1022	1715	84.76309544	138.72832	0.71075415	0.441741559
dre-miR-214	ACAGCAGGCACAGACAGGCAG	956	670	79.28915777	54.1970696	-0.548908753	0.46579662
dre-miR-34a	TGGCAGTGTCTTAGCTGGTTGT	944	986	78.29389638	79.75867258	0.026741557	0.964866719
dre-miR-10b	TACCCTGTAGAACCGAATTTGTG	911	1266	75.55692754	102.4081942	0.438695215	0.656154884
dre-miR-125a	TCCCTGAGACCCTTAACCTGTG	894	1867	74.1469739	151.0237746	1.026315961	0.254177517
dre-miR-216b	TAATCTCTGCAGGCAACTGTGA	760	1099	63.03322166	88.89937238	0.496060832	0.607910708
dre-miR-18b-5p	TAAGGTGCATTTAGTGCAGATA	677	655	56.14933035	52.98370237	-0.083720157	0.860230424
dre-miR-141	TAACACTGTCTGGTAACGATGC	650	1543	53.90999221	124.8150424	1.211167208	0.175725215
dre-miR-18a	TAAGGTGCATCTAGTGCAGATA	594	606	49.26543903	49.02003609	-0.007204368	0.933588196
dre-miR-142a-5p	CATAAAGTAGAAAGCACTACT	579	697	48.02136229	56.38113062	0.231536078	0.845226815
dre-miR-155	TTAATGCTAATCGTGATAGGGG	568	538	47.10903935	43.51943798	-0.114343987	0.832193239
dre-miR-454a	TAGTGCAATATTGCTAATAGGG	521	426	43.21093222	34.45962933	-0.326489172	0.641556946

dre-miR-218a	TTGTGCTTGATCTAACCATGTG	507	371	42.04979392	30.01061615	-0.486625791	0.513924933
dre-miR-139	TCTACAGTGCATGTGTCT	472	445	39.14694819	35.99656116	-0.121040754	0.827846997
dre-miR-216a	TAATCTCAGCTGGCAACTGTGA	466	699	38.64931749	56.54291292	0.54890327	0.567008398
dre-miR-200c	TAATACTGCCTGGTAATGATGC	430	1073	35.66353331	86.79620252	1.283182281	0.151425077
dre-miR-731	AATGACACGTTTTCTCCCGGATCG	388	720	32.18011843	58.24162704	0.855881024	0.350260064
dre-miR-135a	TATGGCTTTTTATTCCTATGTGA	386	312	32.01424153	25.23803838	-0.343114049	0.629398466
dre-miR-135b	TATGGCTTTTTATTCCTATCTG	318	343	26.37442696	27.74566399	0.073122581	0.995849531
dre-miR-20a-3p	ACTGCAGTGTGAGCACTTGAAG	300	246	24.88153487	19.89922257	-0.322363415	0.651747661
dre-miR-27d	TTCACAGTGGCTAAGTTCTTCA	299	389	24.79859642	31.46665683	0.343565441	0.744313489
dre-miR-138	AGCTGGTGTTGTGAATCAGGCC	273	189	22.64219673	15.2884271	-0.566573947	0.456866673
dre-miR-9-5p	TCTTTGGTTATCTAGCTGTATGA	268	289	22.22750448	23.37754196	0.072777262	1
dre-miR-2188-3p	CTGTGTGAGGTTAGACCTATC	246	250	20.40285859	20.22278717	-0.012789451	0.928506931
dre-miR-459-5p	TCAGTAACAAGGATTCATCCTG	237	444	19.65641254	35.91567001	0.869613387	0.342092604
dre-miR-375	TTTGTTCGTTCGGCTCGCGTTA	199	846	16.50475146	68.43391177	2.051830002	0.022521155
dre-miR-137	TTATTGCTTAAGAATACGCGTA	182	123	15.09479782	9.949611286	-0.601339365	0.446287887
dre-miR-27e	TTCACAGTGGCTAAGTTCAGTG	160	219	13.27015193	17.71516156	0.416799734	0.689703974
dre-miR-133a-3p	TTTGGTCCCCTTCAACCAGCTG	160	129	13.27015193	10.43495818	-0.34676007	0.639355422
dre-miR-730	TCCTCATTGTGCATGCTGTGTGT	153	106	12.68958278	8.574461758	-0.565526618	0.469085707
dre-miR-15a-3p	CAGGCCGTACTGTGCTGCGGCA	143	114	11.86019829	9.221590948	-0.363040553	0.625691506
dre-miR-153b	TTGCATAGTCACAAAAATGAGC	140	98	11.61138294	7.927332569	-0.550632403	0.485899294
dre-miR-1788-3p	CAGGCAGCTAAAGCAAGTCTG	125	1120	10.36730619	90.5980865	3.127439502	0.000763782
dre-miR-499	TTAAGACTTGCAGTGATGTTTA	122	143	10.11849085	11.56743426	0.193074769	0.887747885
dre-miR-365	TAATGCCCCTAAAAATCCTTAT	111	215	9.2061679	17.39159696	0.917717753	0.322939509
dre-miR-194b	TGTAACAGCCGCTCCATGTGGA	109	109	9.040291001	8.817135204	-0.03605923	0.913801417
dre-miR-455b	GTATGTGCCCTTGGACTACATT	104	74	8.625598753	5.985945001	-0.527045583	0.496256841
dre-miR-10a-5p	TACCCTGTAGATCCGAATTTGT	92	69	7.630337359	5.581489258	-0.45109673	0.575362704
dre-miR-7a	TGGAAGACTAGTGATTTTGTTGT	88	70	7.298583561	5.662380406	-0.366207832	0.642809275
dre-miR-489	AGTGACATCATATGTACGGCTGC	86	75	7.132706661	6.06683615	-0.233505294	0.739494349
dre-miR-460-5p	CCTGCATTGTACACACTGTGCG	71	61	5.888629918	4.934360069	-0.255069012	0.726601755
dre-miR-142b-5p	CATAAAGTAGACAGCACTACTA	68	63	5.63981457	5.096142366	-0.146242148	0.839626584
dre-miR-218b	TTGTGCTTGATCTAACCATGCA	62	66	5.142183872	5.338815812	0.054138579	1

dre-miR-193b	AACTGGCCCGCAAAGTCCCGCT	60	126	4.976306973	10.19228473	1.034330098	0.27009469
dre-miR-737	AATCAAAACCTAAAGAAAATA	58	87	4.810430074	7.037529934	0.54890327	0.593258738
dre-miR-1306	CCACCTCCCCTGCAAACGTCCA	56	51	4.644553175	4.125448582	-0.17098881	0.806093545
dre-miR-206	TGGAATGTAAGGAAGTGTGTGG	55	50	4.561614725	4.044557433	-0.173562754	0.802659982
dre-miR-19b-5p	AGTTTTGCTGGTTTGCATTCAG	53	58	4.395737826	4.691686623	0.09400131	1
dre-miR-30b	TGTAAACATCCTACACTCAGCT	51	65	4.229860927	5.257924663	0.313883241	0.798106652
dre-miR-17a-3p	ACTGCAGTGGAGGCACTTCTAG	51	47	4.229860927	3.801883987	-0.153895721	0.815482555
dre-miR-9-3p	TAAAGCTAGATAACCGAAAGT	46	44	3.815168679	3.559210541	-0.100189568	0.856753122
dre-miR-187	TCGTGTCTTGTGTTGCAGCC	41	57	3.400476432	4.610795474	0.439278779	0.685578454
dre-miR-204	TTCCCTTTGTCATCCTATGCCT	40	30	3.317537982	2.42673446	-0.45109673	0.614105396
dre-miR-190b	TGATATGTTTGATATTCGGTTG	38	136	3.151661083	11.00119622	1.803476098	0.053314355
dre-miR-129-5p	CTTTTTGCGGTCTGGGCTTGCT	31	33	2.571091936	2.669407906	0.054138579	1
dre-miR-19a-5p	CTAGTTTTGCATAGTTGCACTA	30	27	2.488153487	2.184061014	-0.188062324	0.825562289
dre-miR-153c	TTGCATAGTCACAAAAATGATC	25	29	2.073461239	2.345843311	0.178065575	0.953896618
dre-miR-133a-5p	AGCTGGTAAAATGGAACCAAAT	24	17	1.990522789	1.375149527	-0.53355889	0.551100909
dre-miR-15c	AAGCAGCGCGTCATGGTTTTC	20	9	1.658768991	0.728020338	-1.188062324	0.273747748
dre-miR-184	TGGACGGAGAACTGATAAGGGC	19	50	1.575830541	4.044557433	1.359869446	0.18998496
dre-miR-133b-3p	TTTGGTCCCCTTCAACCAGCTA	19	19	1.575830541	1.536931825	-0.03605923	0.93698066
dre-miR-429b	TAATACTGCCTGGTAATGCCAT	19	69	1.575830541	5.581489258	1.824537713	0.066305698
dre-miR-196a	TAGGTAGTTTCATGTTGTTGGG	18	102	1.492892092	8.250897164	2.46644111	0.012346359
dre-miR-203b-3p	GTGAAATGTTCAGGACCACTTG	13	56	1.078199844	4.529904325	2.070855974	0.047710877
dre-miR-34b	TAGGCAGTGTTGTTAGCTGATTG	11	8	0.912322945	0.647129189	-0.495490849	0.777045532
dre-miR-31	GGCAAGATGTTGGCATAGCTG	10	129	0.829384496	10.43495818	3.65323993	0.000346993
dre-miR-460-3p	CACAGCGCATACAATGTGGATG	9	8	0.746446046	0.647129189	-0.205984232	1
dre-miR-10d-5p	TACCCTGTAGAACCGAATGTGTG	7	5	0.580569147	0.404455743	-0.521486057	0.840434998
dre-miR-20b	CAAAGTGCTCACAGTGCAGGTAG	5	3	0.414692248	0.242673446	-0.773024824	0.2449
dre-miR-212	TAACAGTCTACAGTCATGGCT	5	0	0.414692248	0	-	0.0004998
dre-miR-202-5p	TTCCTATGCATATACCTCTTTG	5	1	0.414692248	0.080891149	-2.357987325	0.06547
dre-miR-183	TATGGCACTGGTAGAATTCACTG	5	9	0.414692248	0.728020338	0.811937676	0.588181503
dre-miR-2187-5p	TTAATTAGTATAGCCTGTTTTA	4	24	0.331753798	1.941387568	2.54890327	0.036013059
dre-miR-132-3p	TAACAGTCTACAGCCATGGTCG	4	5	0.331753798	0.404455743	0.285868865	0.7761

dre-miR-363	AATTGCACGGTATCCATCTGTA	3	2	0.248815349	0.161782297	-0.621021731	0.4523
dre-miR-219	TGATTGTCCAAACGCAATTCTT	3	10	0.248815349	0.808911487	1.700906364	0.239728055
dre-miR-129-3p	AAGCCCTTACCCCAAAAAGCAT	2	3	0.165876899	0.242673446	0.54890327	0.7046
dre-miR-196d	TAGGTAGTTTTATGTTGTTGGG	2	19	0.165876899	1.536931825	3.211868283	0.022314819
dre-miR-203b-5p	AGTGGTTCTCAACAGTTCAACA	2	9	0.165876899	0.728020338	2.133865771	0.178361203
dre-miR-182-5p	TTTGGCAATGGTAGAACTCACA	2	9	0.165876899	0.728020338	2.133865771	0.178361203
dre-miR-459-3p	CAGGGAATCTCTGTTACTGGGG	1	13	0.08293845	1.051584933	3.664380488	0.026706022
dre-miR-457b	AAGCAGCACATAAATACTGGAG	1	0	0.08293845	0	-	0.0004998
dre-miR-7147	TGTACCATGCTGGTAGCCAGT	1	0	0.08293845	0	-	0.0004998
dre-miR-202-3p	AGAGGCATAGGGCATGGGAAAA	1	0	0.08293845	0	-	0.0004998
dre-miR-153a	TTGCATAGTCACAAAAGTGATC	1	11	0.08293845	0.889802635	3.423372388	0.045799632
dre-miR-724	TTAAAGGGAATTTGCGACTGTT	1	3	0.08293845	0.242673446	1.54890327	0.3953
dre-miR-734	GTAAATGCTGCAGAATCGTACCG	0	1	0	0.080891149	-	0.0004998
dre-miR-96	TTTGGCACTAGCACATTTTTGCT	0	2	0	0.161782297	-	0.0004998
dre-miR-1388-5p	AGGACTGTCCAACCTGAGAATG	0	1	0	0.080891149	-	0.0004998
dre-miR-196b	TAGGTAGTTTCAAGTTGTTGGG	0	1	0	0.080891149	-	0.0004998
dre-miR-725	TTCAGTCATTGTTTCTAGTAGT	0	5	0	0.404455743	-	0.0004998
dre-miR-18c	TAAGGTGCATCTTGTGTAGTTA	0	1	0	0.080891149	-	0.0004998
miRNA_chr2_01	UGGCUCAGUUCAGCAGGAAC	712	745	59.05217608	60.26390575	0.029303954	0.994326925
chr20_5274	CACAGCAAGUGUAGACAGGCAG	168	0	13.93365952	0	-	1.19E-08
miRNA_chr6_02	AGCUACAUCCGGCUACUGGGUCU	62	55	5.142183872	4.449013177	-0.208895827	0.821623216
chr4_1432	UUGAGAGGGACGGCCGGG	0	1233	0	99.7387863	-	7.68E-14
chr21_7684	UUAGUGUGAUAAUGGCGUUUGA	0	18	0	1.456040676	-	0.0017497

Supplementary Table 3. The predicted target genes of miR-205.

miRNA	Target gene ID	Names
miR-205	c23998_g2	GRP173
	c30690_g4	GPAT3
	c32585_g1	BCL2
	c33089_g1	KLC2
	c35381_g2	PA24A
	c36330_g1	GPAM
	c36537_g1	ZN521
	c37979_g2	LAMP2
	c38034_g2	APAF1
	c39029_g2	BOC
	c39513_g2	LRP
	c42063_g2	SPSB4
	c42451_g1	CFLAR
	c47003_g5	DHRSX
	c48082_g1	MFTC
	c48385_g1	MOGS
	c48786_g1	RL3
	c49000_g1	EMIL1
	c49090_g1	RBM19
	c49182_g1	RPGF3
	c49436_g1	STIL
	c49470_g1	CNOT1
	c49655_g1	FGD4
	c49886_g2	CASP3

c50723_g2	FKB15
c51130_g2	NAMPT
c51384_g1	CUL9
c51567_g9	LXR
c51784_g8	DPOLZ
c51840_g2	HCDH
c51860_g1	PI4KA
c52157_g3	PEG10
c52293_g6	FASN
c52558_g4	BAZ2B
c55225_g1	SEPT2
c13581_g1	DDIT3
c25984_g1	ACER3
c29321_g1	SYT15
c38628_g1	CAPS2
c39845_g1	EEF1A2
c40029_g1	CDKN2C
c41109_g2	BAX

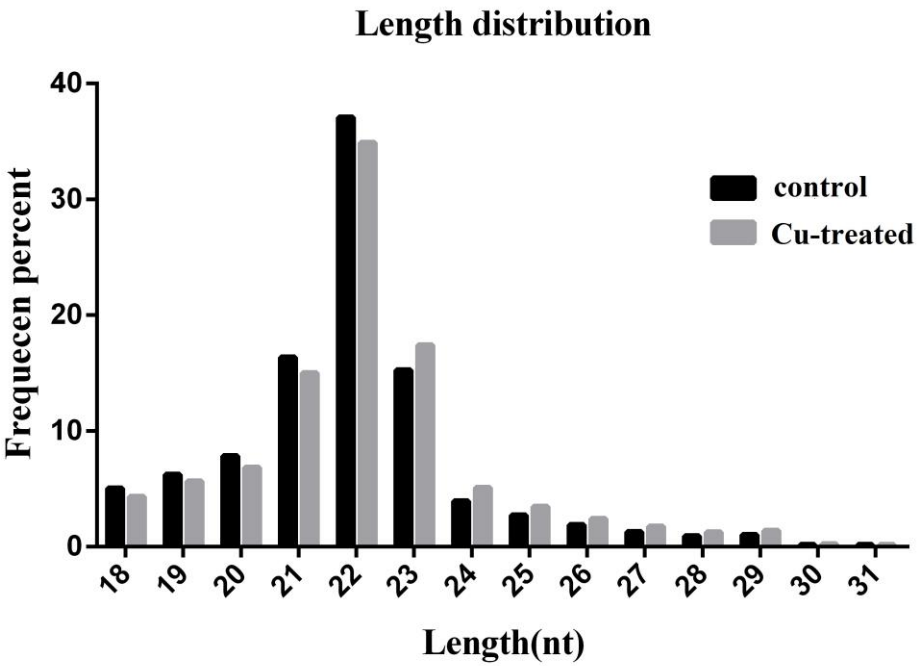

Supplementary Figure 1. Reads length distribution of control and Cu-treated group.

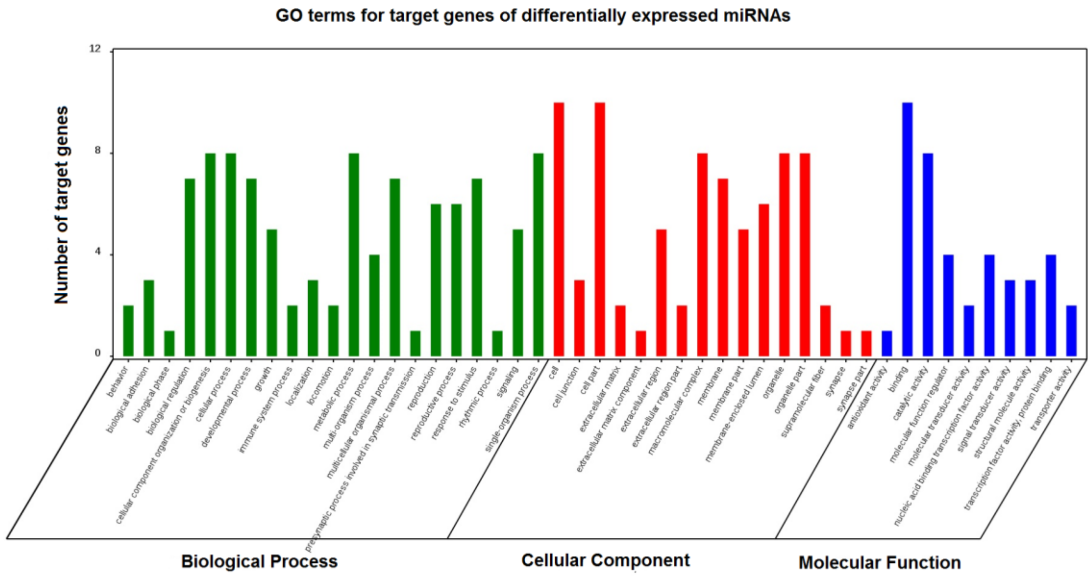

Supplementary Figure 2. GO terms for the target genes of differentially expressed miRNAs.

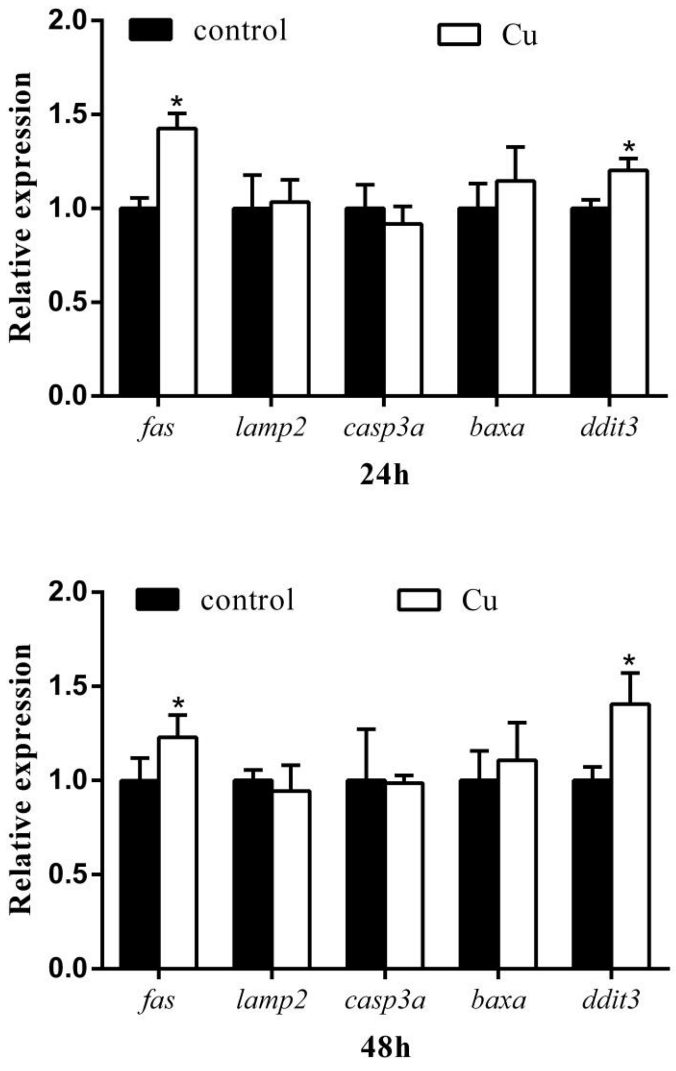

control
Cu
Relative expression
2.0
1.5
1.0
0.5
0.0
*
*
fas
lamp2
casp3a
baxa
ddit3
24h
control
Cu
Relative expression
2.0
1.5
1.0
0.5
0.0
*
*
fas
lamp2
casp3a
baxa
ddit3
48h

Supplementary Figure 3. Effect of Cu incubation on mRNA expression levels of target genes of miRNA-205 from hepatocytes of yellow catfish. Mean of the results from the control was set as 1. Values are mean ± SEM (n = 3). Targets mRNA expression values were normalized to *β-actin* and *tuba* expressed as a ratio of the control at 24 and 48 h. *$p < 0.05$. NS: Student's *T*-test. The experiment was repeated at least three times.
